# A Double Edge‐Sword in Antarctica: In Situ Passive Warming Exacerbates Drought and Heat Stress Differentially in the Native Vascular Species

**DOI:** 10.1111/ppl.70399

**Published:** 2025-07-14

**Authors:** Gago Jorge, Carriquí Marc, Ayuso Manuel, Nunes‐Nesi Adriano, Figueroa Carlos María, Fernie Alisdair Robert, Clemente‐Moreno María José, Gulías Javier, Flexas Jaume, Cavieres Lohegrin Alexis, Bravo León Aloys

**Affiliations:** ^1^ Agro‐Environmental and Water Economics Institute (INAGEA), Research Group of Plant Biology Under Mediterranean Conditions, Department of Biology Universitat de Les Illes Balears Palma Spain; ^2^ National Institute of Science and Technology on Plant Physiology Under Stress Conditions, Departamento de Biologia Vegetal Universidade Federal de Viçosa Viçosa Minas Gerais Brazil; ^3^ Instituto de Agrobiotecnología Del Litoral, UNL, CONICET, FBCB Santa Fe Argentina; ^4^ Central Metabolism Group, Molecular Physiology Department Max‐Planck‐Institut für Molekulare Pflanzenphysiologie Potsdam Germany; ^5^ Departamento de Botánica, Facultad de Ciencias Naturales y Oceanográficas Universidad de Concepción and Instituto de Ecología y Biodiversidad (IEB) Concepción Chile; ^6^ Laboratorio de Fisiología y Biología Molecular Vegetal, Dpt. de Cs. Agronómicas y Recursos Naturales, Facultad de Cs. Agropecuarias y Medioambiente, Instituto de Agroindustria Universidad de La Frontera Temuco Chile

## Abstract

We investigated the impact of open‐top chamber (OTC) passive warming systems at molecular and ecophysiological levels on *Deschampsia antarctica* (DA) and *Colobanthus quitensis* (CQ) in Antarctica. In this field campaign, OTC led to more benign conditions early in the growing season but ultimately intensified drought stress and increased extreme heat events, affecting photosynthetic capacity, metabolism and dehydration tolerance in DA; however, CQ remained relatively unaffected. DA exhibited significant reductions in photosynthesis primarily due to stomatal and mesophyll limitations. Furthermore, DA plants grown under OTC conditions showed a notable 17% decrease in leaf mass per area (LMA), a crucial trait associated with stress tolerance. Metabolic profiling revealed an increased accumulation of osmoprotectants and protein stabilisers (soluble sugars, trehalose, *myo*‐inositol and galactinol), secondary metabolite precursors (tryptophan and nicotinate) and cell wall constituents (xylose) in OTC‐grown DA, suggesting a robust metabolic response to stress. However, these metabolic adjustments were insufficient to counteract the decline in LMA and maintain dehydration tolerance. This study thereby provides new insights into the physiological and metabolic limitations of Antarctic vascular plants under future warming and drying scenarios.

## 1 Introduction

Tundra ecosystems are among the terrestrial biomes most threatened by global warming in recent decades (Henry et al. 2022). Specifically, the southern high latitudes, such as maritime Antarctica and the Antarctic Peninsula, have experienced notable temperature increases, averaging 0.1°C per decade over the past 50 years (Steig et al. 2009; Turner et al. 2016; Jones et al. 2019; González‐Herrero et al. 2022). Annual temperatures are projected to rise by 0.5°C–1.5°C across the Peninsula, with larger increases in autumn and winter (~2°C) over the next two decades (Bozkurt et al. 2021).

Open‐top chambers (OTC) allow researchers to simulate the predicted warming effects driven by climatic change under field conditions, providing valuable information about plant community performance when exposed to increased temperatures (De Alencar et al. 2024). However, OTCs not only increase temperature but also significantly affect the environmental conditions by altering radiation, vapour pressure deficit (VPD), soil moisture and wind sheltering, among other factors, which need to be well documented to understand plant behaviour under those conditions (Henry et al. 2022; Hollister et al. 2023).

Photosynthesis, like all biological processes, is primarily affected by temperature but also by several other factors, like water availability. These factors significantly impact the complex interaction between diffusive (stomatal and mesophyll conductances) and photobiochemical (mainly related to Rubisco activity) processes (Salvucci and Crafts‐Brandner 2004; Yamori et al. 2010, 2014; Flexas et al. 2014; Fernández‐Marín, Gulías, et al. 2020; Moore et al. 2021). The two native Antarctic vascular species, *Colobanthus quitensis* (CQ) and *Deschampsia antarctica* (DA), maintain significant photosynthetic rates at low temperatures, with approximately 30% of their maximum photosynthesis at 0°C (Xiong et al. 1999), although their optimal temperature range for photosynthesis is between 10°C and 23°C (Edwards and Smith 1988; Xiong et al. 1999; Sáez, Galmés, et al. 2018; Clemente‐Moreno et al. 2020a, 2020b; Gago et al. 2023). Leaf temperatures in Antarctic field conditions rarely reach these levels, suggesting that low temperatures significantly limit photosynthesis during the growing season (Xiong et al. 1999; Sáez, Cavieres, et al. 2018). In this sense, temperature increases in the last decade have been linked to improved growth and dominance of both vascular species in the Antarctic tundra, particularly in the northern latitudes of the continent (Cannone et al. 2022; Ramírez et al. 2024). However, in Antarctica, terrestrial primary productivity is not just constrained by low temperatures but also by water and nutrient availability (Crawford 2008; Fernández‐Marín, Gulías, et al. 2020; Gago et al. 2023).

Both CQ and DA exhibit characteristic xerophytic leaf adaptations, including high leaf mass per area (LMA), thick cell walls, enhanced photoprotection, and a high Rubisco specificity factor, which collectively improve water use efficiency (WUE) and dehydration stress tolerance (Sáez, Cavieres, et al. 2018). These adaptations are complemented by hydraulic mechanisms that prevent xylem vessel collapse during freezing and drought (Gago et al. 2014; Nardini 2022; Ramírez et al. 2024). Further, both species showed higher levels of the water–water cycle, non‐photochemical quenching, high levels of superoxide dismutase, ascorbate peroxidase, anti‐freezing proteins, nutrient mobilisation, unsaturated lipids (to maintain membrane fluidity and functionality), respiratory metabolism and important non‐enzymatic antioxidant biochemistry (Bravo et al. 2001; Pérez‐Torres, Dinamarca, et al. 2004; Pérez‐Torres, García, et al. 2004; Clemente‐Moreno et al. 2020a, 2020b; Gago et al. 2023), all of which help to alleviate oxidative stress when photosynthesis (the major electron sink) is impaired in these extreme environments (Fernández‐Marín, Gulías, et al. 2020).

For both species, Sáez, Cavieres, et al. 2018; Sáez, Galmés, et al. 2018 reported that in situ warming reduced some xerophytic traits, such as LMA and leaf density, whereas increasing chloroplast surface exposed to mesophyll airspaces and decreasing the distance between chloroplast and cell wall. Overall, these changes promoted higher mesophyll conductance (*g*_m_) and net photosynthesis (*A*_N_). Nevertheless, a key question that remains unanswered is whether these morphological and biochemical rearrangements, induced by benign temperatures, will ultimately affect their stress tolerance. Although these changes may enhance photosynthetic capacity, it is unclear if they will compromise the xerophytic traits that are essential for survival in the harsh Antarctic environment. Understanding this relationship is crucial for predicting the future development of these species in the tundra under changing climatic conditions. Here, we examined the response of CQ and DA to increased temperatures, including extreme peak events during their growing season at field conditions, as imposed by OTCs on King George Island. This knowledge will provide new insights into how Antarctic ecosystems may respond to climate change.

## 2 Material and Methods

### 2.1 Study Site and In Situ Warming System

**FIGURE 1:**
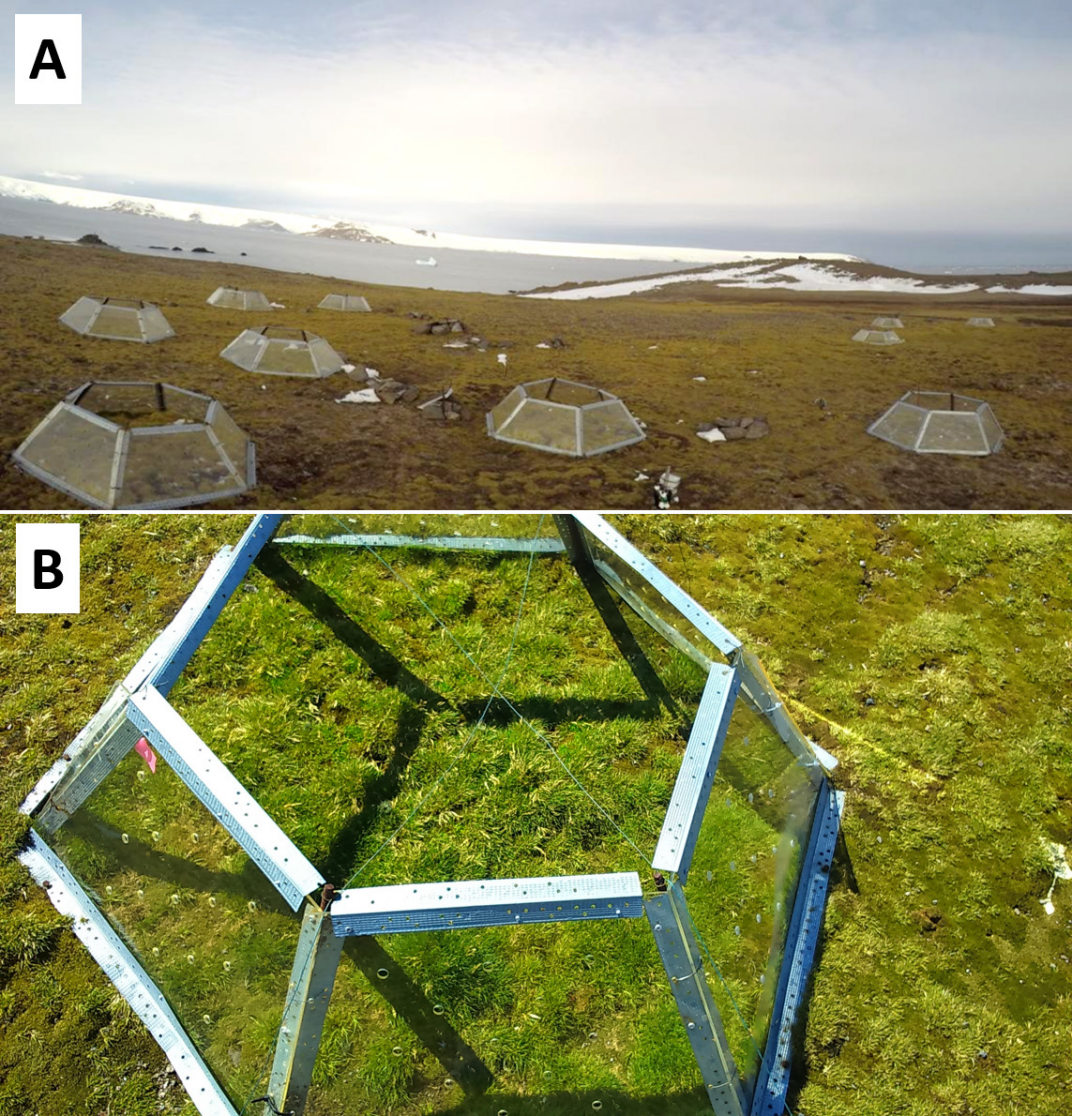
(A) Picture of the experimental site where the open‐top chambers (OTC) and open space (OS) were located at Puchalski Hill in the vicinity of Henryk Arctowski station (King George Island, maritime Antarctica). (B) Top view of *Deschampsia antarctica* and *Colobanthus quitensis* plants growing within the moss carpet in the OTC and OS.

The experimental site was located in the vicinity of Henryk Arctowski Polish Antarctic Station (62°09′ S, 58°28′ W) on Puchalski Hill in the South Shetland Islands, maritime Antarctica. At this location, both perennial native vascular species, *Deschampsia antarctica* Desv. (DA) and *Colobanthus quitensis* (Kunth) Bartl. (CQ), are abundant and intermixed with dominant bryophyte vegetation, forming a continuous moss carpet (Figure 1).

The dominant species in the moss carpet are *Sanionia georgico‐uncinata* (Müll.Hal.) Ochyra and Hedenäs, *Polytrichum piliferum* Hedw., and *Polytrichastrum alpinum* (Hedw.) G.L.Sm. (Kozeretska et al. 2010). DA and CQ individuals are dispersed within the moss, with DA being more abundant (70–200 individuals per m^−2^) and larger in size (ca. 10 cm^2^) compared to CQ (20–150 individuals per m^−2^, ca. 2 cm^2^) (Cavieres et al. 2018). Precipitation at the site primarily falls as snow (ca. 700 mm annually), and the growing season is characterised by cloudy and windy conditions, although brief periods of clear skies can result in high radiation intensities (ca. 2000 μmol m^−2^ s^−1^) (Angiel et al. 2010).

Ten similar 1 m^2^ vegetation plots were selected for the installation of hexagonal open‐top chambers (OTCs), identical to those used in the International Tundra Experiment (ITEX) for passive warming in alpine and Arctic tundras (Hollister et al. 2023). These OTCs were installed in December 2012, as described previously by Sáez, Cavieres, et al. (2018); Sáez, Galmés, et al. (2018). The chambers were constructed using Plexiglass to form 40 cm‐high walls with a basal diameter of 115 cm. To facilitate airflow and prevent excessive heat, each chamber wall was perforated with 25 holes of 1.5 cm in diameter.

Open spaces (OS), located 2 m away from the OTCs to avoid their influence, were selected as control. Microclimate conditions, including air temperature, relative humidity (RH), and photosynthetically active radiation (PAR), were monitored at 5 cm above ground level every hour using two HOBO‐U‐30 stations (Onset Computer Corp.).

From 1st–5th February 2017, individuals from both species in the OTC and OS plots were randomly selected for gas‐exchange measurements, leaf dehydration tolerance assessments, and metabolomics sampling. Leaf temperatures were measured using a T‐type thermocouple Gauge 30, connected to a thermocouple interface SmartFlex‐Hobo (Onset Computer Corp.) in one individual per species per condition. Soil water content (SWC) was recorded using a soil probe Hobo‐10HS Smart sensor (Onset Computer Corp.) at a depth of 10 cm with a frequency of 1 reading/h.

### 2.2 Gas‐Exchange Measurements

Photosynthesis was measured in five individuals of DA and CQ from both the OTC and OS plots during the first week of February 2017, between 08:00 and 16:00. On most occasions, the leaves did not fully cover the measurement chamber area. Therefore, a picture was taken to precisely calculate the leaf area inside the chamber using ImageJ software (Schneider et al. 2012). Leaves were carefully positioned in the gas‐exchange measurement chamber (2 cm^2^; Li‐6400‐40), ensuring no overlap and good contact with the leaf thermocouple of the open gas‐exchange system (equipped with a fluorometer) (Li‐6400XT; Li‐Cor Inc.).

**FIGURE 2:**
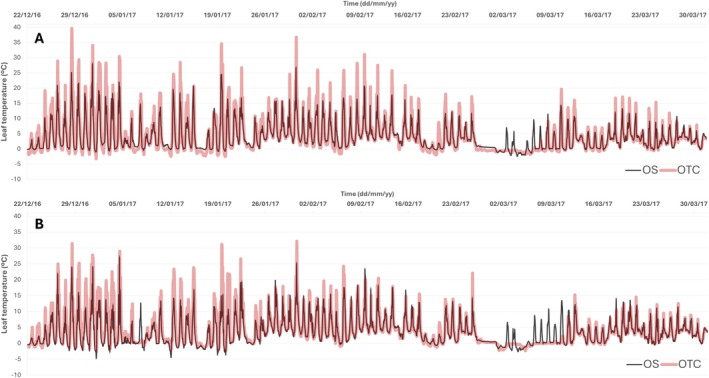
Leaf temperature for *Deschampsia antarctica* (A) and *Colobanthus quitensis* (B) recorded throughout the 2016–2017 growing season in plants located inside the open‐top chamber (OTC) and outside (OS).

Gas‐exchange measurements were conducted at two block temperatures, 15°C and 5°C, imitating optimal and suboptimal temperatures for photosynthesis (Sáez, Cavieres, et al. 2018; Sáez, Galmés, et al. 2018 and references therein) that can be frequently reported under field conditions during the growing season (Figures 2 and S1). Once steady‐state conditions were reached, net photosynthesis (A_N_), stomatal conductance (g_s_), substomatal CO_2_ concentration (C_i_), and maximum fluorescence under light conditions (Fm′), using a saturating pulse (8000 μmol quanta, 0.8 s duration) were recorded. Chamber conditions were set to 400 ppm CO_2_ of C_a_, 2000 μmol m^−2^ s^−1^ of PPFD (90%:10% red:blue light) and 50%–70% relative humidity (VPD ranging from 1.5 to 2.5 kPa). Additionally, leaves were kept in darkness for 30 min to measure respiration (*R*_d_). Mitochondrial respiration under light conditions was considered as half of *R*_d_ (Niinemets et al. 2005).

Electron transport rate (ETR) was calculated as ETR = ΦPSII * PAR * α * β (Genty et al. 1990), where leaf absorbance (α) was measured using a spectrometer (HR2000CG‐UV‐NIR; Ocean Optics Inc.), following the same Li‐COR led light settings previously described. The partitioning of energy between PSII/PSI (β) was determined for each temperature as described by Clemente‐Moreno et al. (2020a, 2020b). Photorespiration was calculated following the model proposed by Valentini et al. (1995), combining gas‐exchange and chlorophyll fluorescence. This model assumes that all reducing power produced through electron transport is employed for either photosynthesis or photorespiration, with chlorophyll fluorescence measurements as a reliable proxy for the quantum yield of electron transport. Thus, following the known stoichiometries of electron use in photorespiration, it can be solved as follows: *P*_r_ = 1/12 [ETR−4 (*A*_area_ + *R*_d_/2)].

Mesophyll conductance to CO_2_ (*g*_m_) was calculated by employing the variable *J* method (Harley et al. 1992), where *g*_m_ = *A*_area_/{C_i_−Γ* [ETR + 8 (*A*_area_ + *R*_d_/2)]/[ETR−4 (*A*_area_ + *R*_d_/2)]}. Γ* is the CO_2_ compensation point without mitochondrial respiration, derived from the Rubisco kinetics at both 15°C and 5°C determined by Sáez, Cavieres, et al. (2018); Sáez, Galmés, et al. (2018). The CO_2_ concentration at the chloroplast stroma (*C*_c_) was calculated as *C*_c_ = *C*_i_−*A*_area_/*g*_m_.

Finally, relative photosynthetic limitations, including stomatal (l_s_), mesophyll (l_m_) and photobiochemical (l_b_) limitations, were estimated following the Grassi and Magnani (2005) model.

### 2.3 Leaf Dry Mass per Area and Relative Water Content

Leaf mass per area (LMA) was calculated as the ratio of leaf area to dry mass. Leaf area was measured using fresh samples and analysed employing the ImageJ software (Schneider et al. 2012). The samples were then dried in an oven at 65°C until a constant weight was achieved (3–4 days), and their dry weight was recorded. Photosynthesis on an area basis was subsequently transformed to a mass basis using the LMA of the same individual.

Relative water content (RWC) was calculated using the formula RWC = [(FW−DW)/(TW−DW)] × 100, where FW is the fresh weight, TW is the turgid weight, and DW is the dry weight. To determine FW, leaf samples were excised from individuals and immediately kept in humidity‐saturated zip‐lock bags for approximately 30 min until they were weighed in the laboratory. After measuring FW, samples were placed in six well‐plates, covered with distilled water‐soaked paper, and left for 24 h at 4°C to reach full turgor. TW was measured after this period. DW was determined by drying the samples at 65°C, as described for LMA measurements.

### 2.4 Leaf Dehydration Tolerance

Leaf dehydration tolerance was assessed using the ‘Falcon test’ method, as described by López‐Pozo et al. (2019). Photosynthetic tissues (ca. 100 mg per replicate) were collected and initially hydrated by placing them in wet paper for 24 h under dark conditions. After this hydration period, the leaf tissues were transferred to 50 mL Falcon tubes and subjected to dehydration at three different humidity levels (80%, 50% and > 10% RH) using desiccants (NaCl, MgCl_2_ and silica gel, respectively) after the atmosphere reached the equilibrium. Samples were maintained under these dehydration conditions for 48 h. After dehydration, the samples were rehydrated with wet paper for 24 h. The whole process was conducted in darkness and at a temperature of 22°C–25°C.

At each step of the process, samples were weighed, and dry weight was determined by drying them in an oven at 65°C until a constant weight was achieved (3–4 days) to calculate RWC. The maximal photochemical quantum efficiency of PSII (F_v_F_m_) was measured at each step (initial hydration after 24 h under hydration, after desiccation and after rehydration) using a JUNIOR‐PAM fluorometer (Heinz Walz). Chlorophyll fluorescence was used as a proxy for photosynthetic apparatus integrity and, therefore, as an indicator of physiological status (López‐Pozo et al. 2019). Minimum fluorescence (*F*_o_) was estimated under dim measuring light, whereas maximum fluorescence (*F*_m_) was induced by a saturation pulse (8000 μmol quanta m^−2^ s^−1^, 0.8 s duration). The variable fluorescence (*F*_v_) was calculated as the difference between *F*_m_ and *F*_o_, and finally, *F*_v_ *F*_m_ values were recorded. The percentage of recovery of the *F*_v_ *F*_m_ was calculated considering the initial and the rehydrated values following desiccation.

### 2.5 Primary Metabolite Analysis

Leaf samples were collected at midday from 6 to 8 individuals for metabolomic analysis for all species and treatments. They were immediately frozen in liquid nitrogen in the field using a dry dewar shipper Voyager 12 Cryo (AirLiquide) and stored there until our return to continental Chile, where they were kept at −80°C. Prior to extraction, the samples were lyophilised. The metabolite extraction protocol for primary metabolite analysis was conducted as previously described (Lisec et al. 2006). Ribitol was employed as an internal standard (0.2 mg mL^−1^ in H_2_O). An aliquot (150 μL) of the polar phase was dried and re‐suspended in methoxyamine hydrochloride (20 mg mL^−1^ in pyridine) and derivatised using *N*‐methyl‐*N*‐[trimethylsilyl]trifluoroacetamide (MSTFA).

The GC‐TOF‐MS system contains a CTC CombiPAL autosampler, an Agilent 6890 N gas chromatograph, and a LECO Pegasus III TOF‐MS running in EI+ mode. Two sample injection volumes were employed (1 and 10 μL of the derivatised metabolite extraction) to cover a broader range of metabolite concentrations in our samples. Chromatogram peaks were annotated to metabolites by comparing mass spectra and GC retention times with the library from the Golm Metabolome Database (Kopka et al. 2005) using TagFinder (v4.0) and Xcalibur 2.1 software (Thermo Fisher Scientific).

### 2.6 Statistics

Two‐way ANOVA followed by Tukey's HSD test (*p* < 0.05) for multiple comparisons was performed for gas‐exchange and dehydration test measurements; for the rest of the analysis, one‐way ANOVA was employed. R software (v. 4.1.1.) with the packages ‘dplyr’, ‘dlookr’, ‘writexl’ and ‘agricolae’ was used for statistical analysis.

The Thompson τ method was used to detect outliers in the metabolomics dataset. The data were then normalised by subtracting the mean and dividing by the standard deviation of each metabolite. A multivariate analysis (PLS‐DA) was conducted using the online software platform MetaboAnalyst 6.0 (https://www.metaboanalyst.ca) to explore the discriminant features associated with different metabolites between samples. Fold changes in metabolites between the OTC and control samples were visualised as a heatmap using GraphPad Prism software (Prism version 10.2.3).

## 3 Results

### 3.1 Warmer but Drier: Microclimate Effects in OTCs

From 1 December 2016 to 31 March 2017, the average nocturnal air temperature at OS was 0.60°C ± 0.07°C, whereas the diurnal average temperature was 3.40°C ± 0.06°C. The lowest air temperature was recorded on the night of March 4th at −6.9°C, whereas the highest temperature (12.4°C) occurred on 9th February. Inside the OTCs, the average temperature was higher, especially during the daytime, with an average of 4.60°C ± 0.08°C. The maximum air temperature in OTCs was significantly higher (20.7°C, January 2017) compared to the OS, whereas minimum temperatures remained similar between treatments (around −6°C; Figure S1).

Leaf temperatures followed a similar pattern to air temperatures, showing clear differences between inside and outside the OTCs for both species (Figure 2). In DA, the average diurnal leaf temperature inside the OTCs was 6.82°C, with maximum values reaching up to 39.7°C during specific periods, whereas the minimum temperature was −3.2°C. In contrast, the average diurnal leaf temperature in the OS was slightly lower (5.8°C), with maximum values ranging between 25°C and 26°C, considerably lower values than OTC. The absolute minimum temperature at OS was −3°C, similar to OTC (Figure 2A). CQ showed the same trend as DA, with OTC plants showing a higher average leaf temperature (6.2°C) than OS (5.6°C). There were important differences regarding the maxima, in this case, lower than in DA, but still reaching up to 32.5°C, whereas OS plants showed even lower maximum values (ca. 25°C). Minimum absolute values were similar for both treatments in CQ, with values of −3°C (Figure 2B).

**FIGURE 3:**
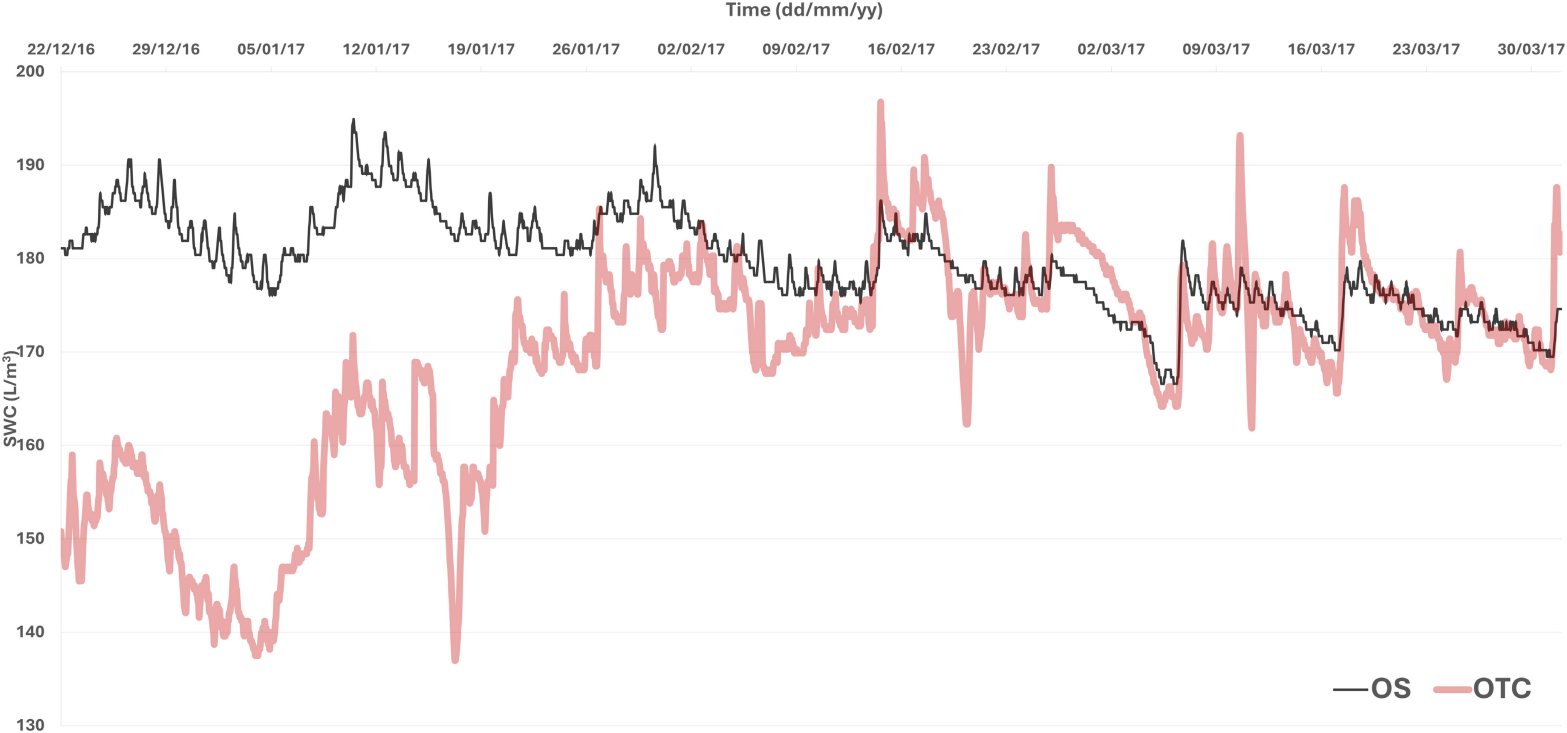
Soil water content (SWC, L/m^3^) at a depth of 10 cm recorded throughout the 2016–2017 growing season, comparing conditions inside the open‐top chamber (OTC) and outside (OS).

Another relevant result of the microclimate effect of OTCs in this growing season was the reduced soil water content at the beginning of the summer inside the OTCs (Figure 3), just increasing to similar levels to OS at the end of the growing season (at the beginning of February). From this point, both treatments displayed similar values for this parameter up to the end of the summer season (Figure 3).

### 3.2 Photosynthesis Was Constrained by Drought Rather Than Favoured by the In Situ Warming of the OTCs

Leaf traits such as leaf absorbance, LMA, and RWC were analysed for both species under the different environmental conditions (Table S1). In DA, a significant reduction in LMA was observed in plants growing within the OTCs compared to those in OS. CQ exhibited no significant differences in leaf absorbance or LMA; however, RWC was significantly higher in OTC plants compared to those in OS (Table S1).

Net photosynthesis (*A*_N_) was measured at two different temperatures (15°C and 5°C) for both species and treatments. At 15°C, *A*_N_ ranged from 4.4 to 0.9 μmol CO_2_ m^−2^ s^−1^ in DA and from 6.9 to 6.2 μmol CO_2_ m^−2^ s^−1^ in CQ. When measured at the suboptimal temperature of 5°C, *A*_N_ ranged from 1.4 to 0.4 μmol CO_2_ m^−2^ s^−1^ in DA and from 5.9 to 4.7 μmol CO_2_ m^−2^ s^−1^ in CQ (Table 1). Compared to previous field campaigns at the same location, these values were extremely low for DA and within a similar range for CQ (Sáez, Galmés, et al. 2018; Gago et al. 2023).

**TABLE 1:** Photosynthetic characterisation of *Deschampsia antarctica* and *Colobanthus quitensis* grown in open space (OS) and inside open‐top chambers (OTC), measured at 15°C and 5°C.

*T* (°C)	Growing conditions	*A*_N_	*A*_N_mass_	*g*_s_	*C*_i_	ETR	*P*_r_	*g*_m_	*C*_c_	ETR/*A*_N_
*Deschampsia antarctica*										
15	OS	4.4 ± 1.5	0.05 ± 0.02	0.025 ± 0.007	214.5 ± 32.2	60.3 ± 2.1a	3.2 ± 0.6a	0.012 ± 0.002	49.51 ± 1.0	20.3 ± 5.2
15	OTC	0.9 ± 0.6	0.01 ± 0.01	0.013 ± 0.003	297.1 ± 86.7	42.3 ± 3.9b	2.9 ± 0.2ab	0.007 ± 0.005	46.49 ± 6.3	84.2 ± 56.2
5	OS	1.4 ± 0.1	0.02 ± 0.00	0.026 ± 0.007	331.0 ± 13.0	25.0 ± 5.6c	1.2 ± 0.42b	0.007 ± 0.002	84.9 ± 34.3	16.9 ± 2.3
5	OTC	0.4 ± 0.5	0.01 ± 0.01	0.014 ± 0.001	378.4 ± 35.0	19.6 ± 1.8c	1.4 ± 0.23b	0.005 ± 0.0006	48.6 ± 12.0	15.6 ± 4.3
*p*	Gr. Cond.	**0.021**	**0.033**	**0.048**	0.220	**< 0.001**	0.636	0.324	0.353	0.235
	*T*	0.125	0.129	0.851	0.123	**< 0.001**	**0.001**	0.340	0.338	0.398
	Gr. Cond.*T	0.260	0.302	0.984	0.772	**0.087**	0.648	0.702	0.438	0.375
*Colobanthus quitensis*										
15	OS	6.2 ± 1.6	0.07 ± 0.02	0.19 ± 0.02a	358.5 ± 9.7a	134.6 ± 15.0a	8.2 ± 0.8a	0.022 ± 0.006	63.4 ± 4.3	33.5 ± 14.9
15	OTC	6.9 ± 1.3	0.08 ± 0.02	0.09 ± 0.01b	310.7 ± 8.3b	89.9 ± 6.5b	4.30.3ab	0.036 ± 0.010	99.1 ± 13.2	14.7 ± 2.3
5	OS	5.9 ± 1.0	0.07 ± 0.01	0.13 ± 0.01ab	346.2 ± 7.0ab	62.5 ± 8.0bc	2.6 ± 0.8b	0.024 ± 0.007	51.7 ± 19.5	12.4 ± 3.3
5	OTC	4.7 ± 1.1	0.06 ± 0.01	0.09 ± 0.02b	332.7 ± 15.3ab	45.6 ± 3.7c	1.8 ± 0.4c	0.026 ± 0.009	108.4 ± 32.4	11.4 ± 2.1
*p*	Gr. Cond.	0.874	0.856	**< 0.001**	**0.010**	**0.004**	**0.001**	0.334	**0.040**	0.192
	*T*	0.354	0.363	0.127	0.653	**< 0.001**	**< 0.001**	0.639	0.978	0.114
	Gr. Cond.*T	0.466	0.461	**0.048**	0.124	0.154	**0.021**	0.509	0.615	0.240

*Note:* Values are presented as means ± SE (*n* = 5–6). Parameters include net photosynthesis on an area basis (*A*_N_; μmol CO_2_ m^−2^ s^−1^) and mass basis (*A*_N_mass_; μmol CO_2_ g^−1^ s^−1^), stomatal conductance to CO_2_ (*g*_s_; mol m^−2^ s^−1^), CO_2_ concentration in substomatal cavities (*C*_i_; μmol mol^−1^), electron transport rate (ETR; μmol m^−2^ s^−1^), photorespiration (*P*_r_; μmol m^−2^ s^−1^), mesophyll conductance to CO_2_ diffusion (*g*_m_; mol m^−2^ s^−1^), CO_2_ concentration at Rubisco carboxylation sites in the chloroplast stroma (*C*_c_; μmol mol^−1^), and the electron transport rate to photosynthesis ratio (ETR/*A*_N_; μmol e^−^/μmol CO_2_). Significant differences between treatments are highlighted in red, with *p*‐values indicating the effects of growing conditions, temperature (*T*) and their interaction. Different letters represent significant differences between groups by multiple comparison's Tukey's test (*p* < 0.05).

For DA, *A*_N_, *A*_N_mass_ and *g*_s_ were significantly affected by OTCs, regardless of the measurement temperature, with higher values observed in OS compared to OTC (Table 1). Only ETR was significantly affected interactively by both the growing condition and measurement temperature, with plants from inside the OTC showing lower ETR values (Table 1). Photorespiration (*P*_r_) was the only parameter significantly affected by the measuring temperature alone (Table 1). The ETR/*A*_N_ ratio, used to assess physiological status (Perera‐Castro and Flexas 2023), indicated values ranging from 84.2 to 20.3 μmol e^−^/μmol CO_2_ under optimal temperature conditions (15°C)—much higher than the typical range for C_3_ species at optimal conditions (7.5–10.5 μmol e^−^/μmol CO_2_)—suggesting that DA was under severe physiological stress. However, no significant differences between OS and OTC were observed for this parameter (Table 1).

The response of CQ was different from that of DA. *A*_N_ showed no significant effects, but *g*_s_ was lower in OTC plants compared to OS, with a significant interaction between growing conditions and temperature (Table 1). Both *C*_i_ and *C*_c_ were lower in OTC plants, though unaffected by the measurement temperature (Table 1). ETR and *P*_r_ were strongly influenced by both the measurement temperature and the growing site, with *P*_r_ showing a significant interaction between these factors, leading to a significant reduction associated with the lower measurement temperature (Table 1). The ETR/*A*_N_ ratio also indicated suboptimal physiological conditions for CQ but to a much lesser extent than for DA, with values ranging from 14.7 to 33.5 μmol e^−^/μmol CO_2_ at optimal temperature (Table 1). No significant differences in ETR/*A*_N_ were observed between OTC and OS (Table 1). Overall, the OTC environment significantly affected *g*_s_ and photobiochemistry (as reflected by ETR) for both species during the summer season (Figures 2, 3 and S1). However, *g*_m_ was not significantly affected by any of the conditions tested. This could be explained by the lower values already seen in both conditions compared with previous field campaigns (Sáez, Cavieres, et al. 2018; Sáez, Galmés, et al. 2018; Gago et al. 2023), probably due to the prolonged water limitation imposed from the beginning of the growing season (Figure 3). In fact, water limitation could become more severe in DA during this field campaign because the individuals within OTCs exhibited bigger size than those outside, with this observation being particularly prominent in the case of DA, as observed previously (Bravo, pers. comm.; Figure S2).

### 3.3 Leaf Dehydration Tolerance of *Deschampsia antarctica* Is Reduced in OTCs

We used the leaf dehydration tolerance test of López‐Pozo et al. (2019) to assess whether the microclimate of OTCs affected the dehydration tolerance of DA and CQ. Initial *F*_v_ *F*_m_ values obtained for both species were within the optimal range for angiosperms (López‐Pozo et al. 2019) and showed no differences between plants from OTC and OS (Table 2). Leaf dehydration was significant across all treatments, with RWC decreasing by 11.1%–46.2% in DA and 6.0%–27.9% in CQ.

**TABLE 2:** Dehydration tolerance test for *D. antarctica* and *C. quitensis* individuals grown in open space (OS) and inside open‐top chambers (OTC).

Species	Growing conditions	Dessicant	*F*_v_ *F*_m_ initial	*F*_v_ *F*_m_ dehydrated	RWC dehydration (%)	*F*_v_ *F*_m_ Recovery (%)
*Deschampsia antarctica*	OS	NaCl	0.78 ± 0.003	0.72 ± 0.02a	46.2 ± 5.6a	93.0 ± 0.8a
		MgCl_2_	0.79 ± 0.006	0.69 ± 0.02ab	38.7 ± 3.6a	87.8 ± 1.9a
		Silica	0.77 ± 0.008	0.68 ± 0.01ab	11.1 ± 4.2b	92.1 ± 2.2a
	OTC	NaCl	0.75 ± 0.170	0.70 ± 0.01ab	38.6 ± 2.3a	95.5 ± 1.4a
		MgCl_2_	0.79 ± 0.007	0.59 ± 0.03bc	19.4 ± 2.1b	88.4 ± 3.4a
		Silica	0.78 ± 0.004	0.50 ± 0.04c	13.1 ± 2.3b	73.1 ± 4.7b
	F‐statistic		2.29	12.79	17.38	8.59
	*p*	Grow. cond	0.492	**< 0.001**	**0.003**	**0.034**
		Desiccant	0.110	**< 0.001**	**< 0.001**	**0.004**
		Grow. conditions: Desiccant	0.100	**0.023**	**0.041**	**0.003**
*Colobanthus quitensis*	OS	NaCl	0.79 ± 0.01	0.69 ± 0.02a	27.9 ± 3.8a	89.2 ± 3.3ab
		MgCl_2_	0.77 ± 0.02	0.62 ± 0.04a	19.7 ± 3.1ab	83.5 ± 4.9ab
		Silica	0.78 ± 0.01	0.62 ± 0.01a	14.8 ± 1.5ab	61.5 ± 7.0c
	OTC	NaCl	0.76 ± 0.01	0.67 ± 0.05a	22.9 ± 5.2b	89.9 ± 1.3a
		MgCl_2_	0.77 ± 0.01	0.58 ± 0.01a	12.4 ± 0.8b	68.5 ± 4.7bc
		Silica	0.73 ± 0.03	0.43 ± 0.02b	6.0 ± 1.6b	81.7 ± 3.5abc
	F‐statistic		1.28	9.4	5.24	6.69
	*p*	Grow. conditions	0.116	**0.004**	**0.045**	0.603
		Desiccant	0.450	**0.001**	**0.003**	**0.004**
		Grow. conditons: Desiccant	0.426	**0.026**	0.852	**0.007**

*Note:* Three desiccation treatments were applied: NaCl (80% RH), MgCl_2_ (45% RH) and Silica gel (< 10% RH). The table includes initial *F*_v_ *F*_m_ values, *F*_v_ *F*_m_ and relative water content (RWC) after 48 h at the desiccation stage, and *F*_v_ *F*_m_ recovery (%) after 24 h of rehydration. Values are presented as means ± SE (*n* = 3). Significant differences between treatments are highlighted in red, with *p*‐values reflecting the effects of growing conditions, desiccant, and their interaction. Different letters represent significant differences between groups by multiple comparison's Tukey's test (*p* < 0.05).

The level of dehydration was influenced not only by the desiccants used but also by the differences between OS and OTC treatments in both species. For DA, a significant interaction between treatment and desiccant was observed (Table 2), indicating that drought imposed by OTCs may induce structural and biochemical changes that affect *F*_v_ *F*_m_ under dehydration and the leaf desiccation level obtained (RWC_dehydration). Dehydration tolerance, assessed by the recovery of the *F*_v_ *F*_m_, also varied depending on the desiccant applied. Additionally, dehydration tolerance in DA differed significantly between the OTC and OS treatments, with higher recovery values obtained in OS than in OTC; these differences were not observed in CQ. In both species, we observed a significant interaction between desiccant and the growing conditions (Table 2). This reduction in dehydration stress tolerance in DA plants grown in OTCs could be attributed to structural factors (e.g., lower LMA; Table S1), as well as biochemical mechanisms, such as increased carbon and/or energy investment in secondary metabolism.

### 3.4 Primary Metabolism Was Affected by OTCs

**FIGURE 4:**
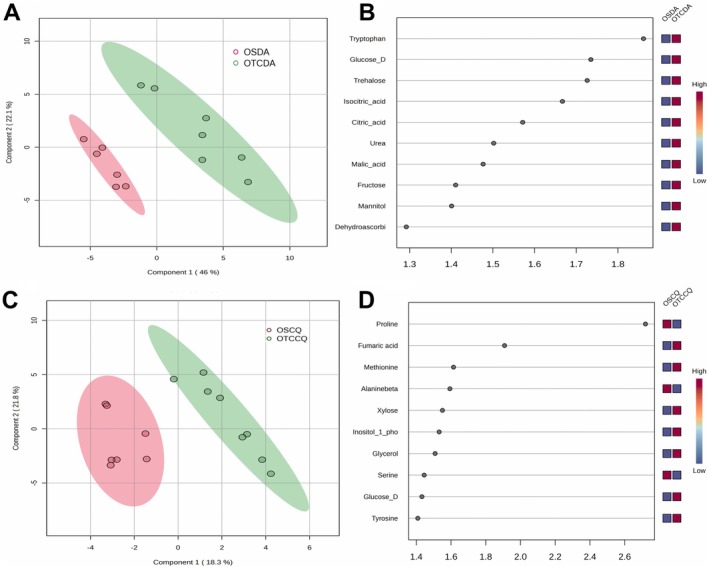
PLS‐DA analysis of the primary metabolic profiles for *Deschampsia antarctica* (DA) (upper panels) and *Colobanthus quitensis* (CQ) (lower panels). (A and C) Score plots illustrating the separation between individuals from open space (OS) and open‐top chambers (OTC) based on metabolic profiles. (B and D) The top 10 VIP metabolites contributing to the separation between treatments.

We annotated 52 primary metabolites in both species (Table S2). To explore the main differences in metabolic profile, we employed a Partial Least Squares Discriminant Analysis (PLS‐DA) modelling. For DA, the model explained 68.8% of the variance (Figure 4a), whereas for CQ, the model explained 40.7% (Figure 4c). These results are consistent with the gas‐exchange data and physiological status, showing more pronounced differences between OTC and OS treatments in DA than in CQ (Tables 1 and 2).

In DA, the major 10 variable importance in projection’ (VIP) metabolites driving the differences between treatments were mainly sugars (glucose, trehalose, and fructose) and TCA cycle intermediates (citrate, isocitrate, and malate). The list also included urea, mannitol, dehydroascorbate, and tryptophan (Figure 4b). It is important to note that all these compounds were increased in DA plants growing in OTCs when compared to OS plants. For CQ, we observed a different pattern, with proline and fumarate emerging as the most important metabolites, although with opposite trends (proline was lower and fumarate was higher in OTC than OS plants). Other significant metabolites included amino acids (methionine, β‐alanine, serine, and tyrosine), sugars (glucose and xylose), inositol 1‐phosphate and glycerol (Figure 4d).

**FIGURE 5:**
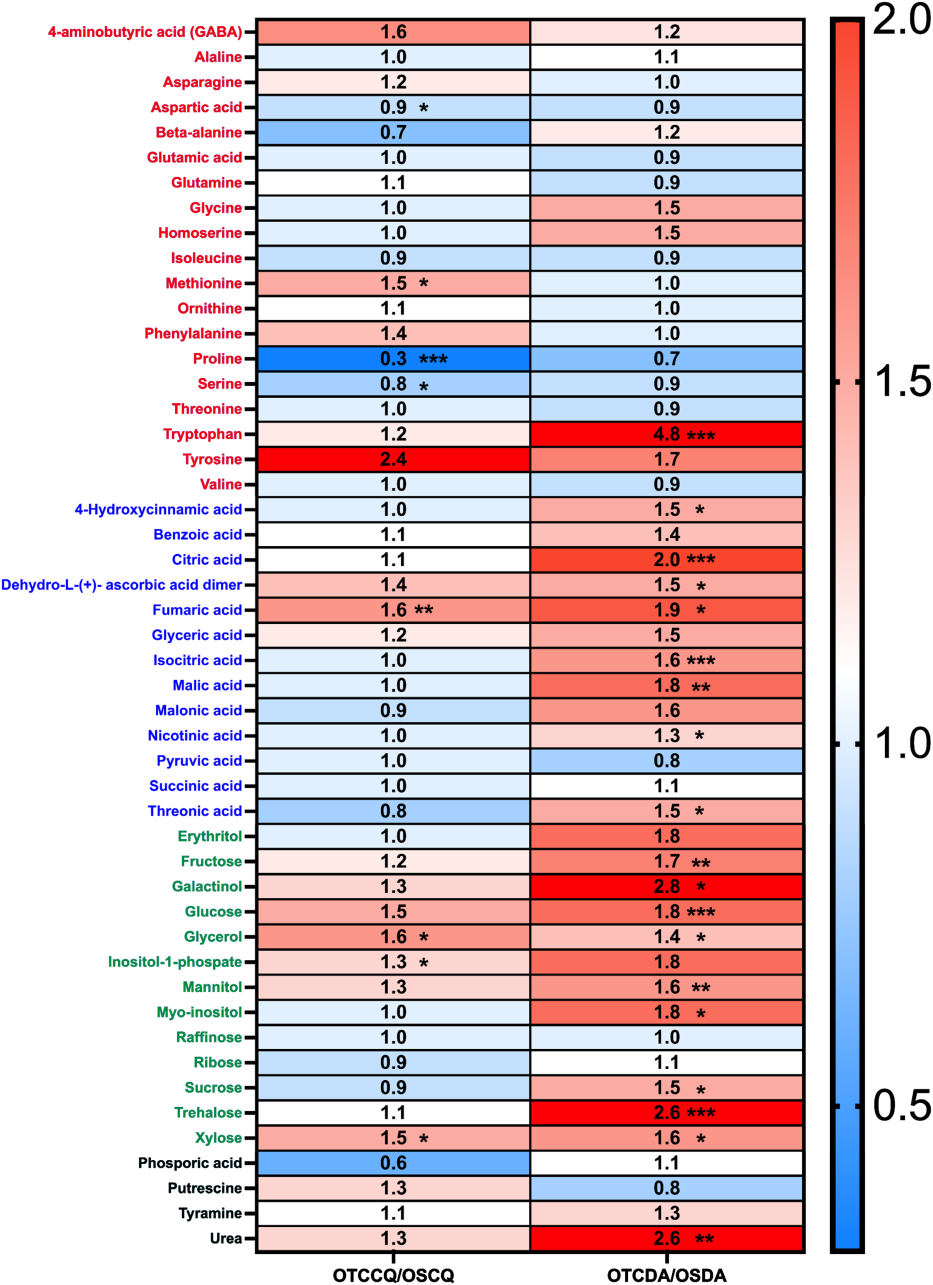
Heatmap of fold changes in metabolite levels for *Colobanthus quitensis* (CQ) and *Deschampia antartica* (DA) grown under open‐top chambers (OTC) and open space (OS) conditions (OTCCQ, OSCQ, OTCDA, and OSDA, respectively). Asterisks indicate significant differences between growth conditions as determined by the *t*‐test (*p < 0.05; **p < 0.01; ***p < 0.001). The colour scale represents fold changes, with red indicating higher levels and blue indicating lower levels of metabolites. The metabolites are sorted and coloured based on their main chemical classes (red for amino acids, blue for organic acids, green for sugars and sugar‐alcohols, and black for ‘other’ classes).

We also used a heatmap to analyse the changes in metabolite levels displayed by plants grown in OTC and OS (Figure 5). Regarding DA plants, all the metabolites that displayed statistically significant differences (i.e., 38% of all the annotated metabolites) were increased in OTC growing conditions compared to OS. The most altered group of metabolites was that containing sugar and sugar alcohols, including glucose, fructose, sucrose, xylose, trehalose, mannitol, glycerol and myo‐inositol (Figure 5). TCA intermediates (citrate, isocitrate, malate and fumarate) were also significantly increased, as well as nicotinate and tryptophan, which are precursors of secondary metabolites (Figure 5). In CQ plants, only 16% of the annotated metabolites were significantly altered. From these, only three were decreased: aspartate, serine and proline. Conversely, methionine, fumarate, glycerol, inositol 1‐phosphate and xylose were significantly increased (Figure 5).

## 4 Discussion

**FIGURE 6:**
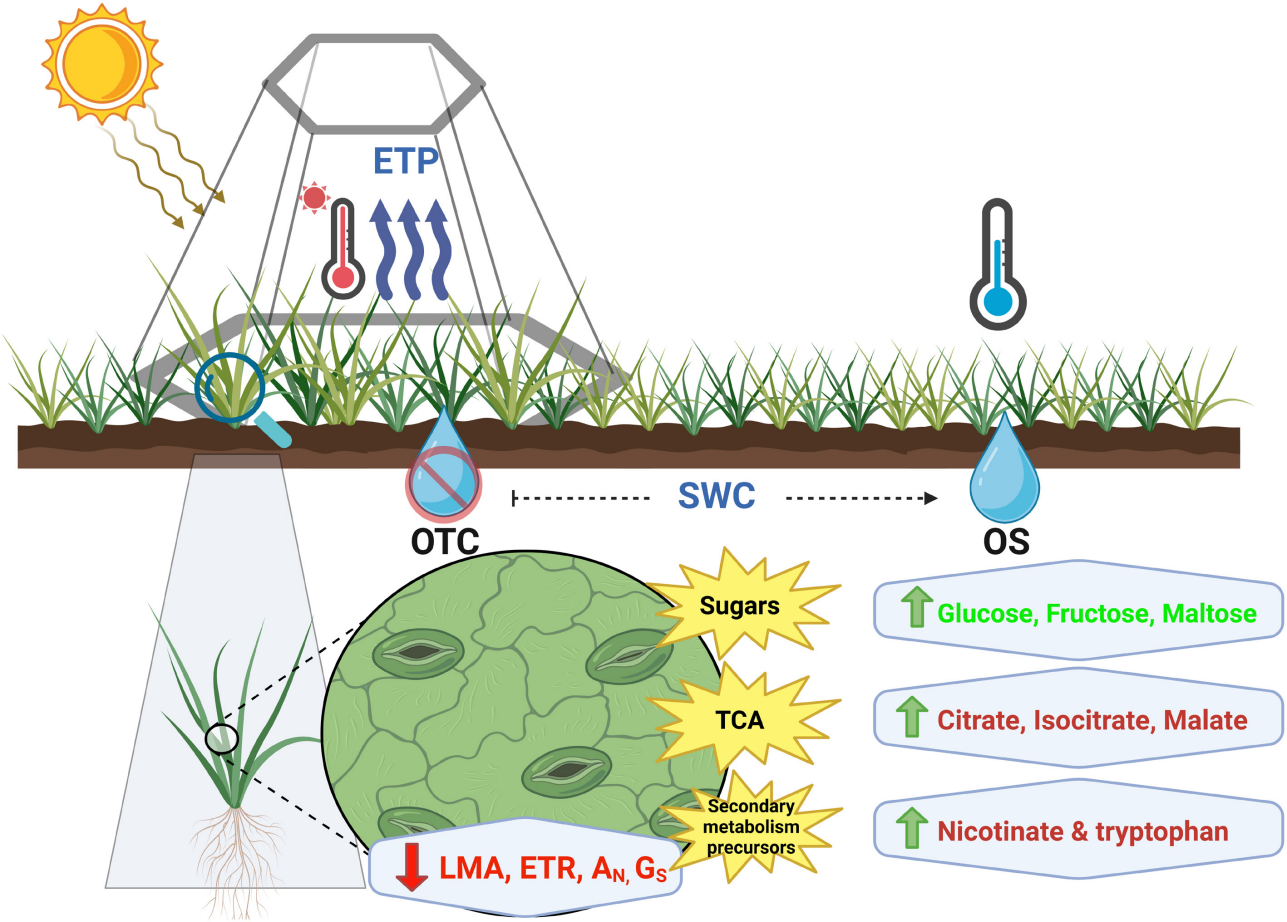
Holistic view of the OTCs environment effect in the molecular ecophysiology response of *Deschampsia antarctica* (DA). Zoom in OTC DA individual shows its responses at leaf level regarding leaf structure, photosynthesis, and its physiological drivers, accompanied by its primary metabolism compared to OS plants. OTC (Open‐Top Chamber), OS (Open Space), ETP (Evapotranspiration), SWC (Soil Water Content), LMA (leaf dry mass per area), ETR (electron transport rate), *A*_N_ (net photosynthesis), *g*_s_ (stomatal conductance).

OTCs are used to study climate change impacts in tundra and high‐elevation ecosystems, particularly in Antarctica, where they simulate warming but also modify critical micro‐environmental factors (VPD, soil moisture, wind sheltering) (Henry et al. 2022; Hollister et al. 2023). This region faces significant climate threats, including rising temperatures and uncertain summer precipitation patterns (Bozkurt et al. 2021; González‐Herrero et al. 2022). During our study at the Arctowski Polish Research Station, typical air temperatures were recorded, but soil moisture was lower than in previous assessments (Sáez, Galmés, et al. 2018) (Figures 2, 3 and S1), offering valuable insights into the effects of climate change in situ. In OTCs, DA leaf temperatures soared to significant values, reaching ca. 40°C during warm, clear‐sky days (21°C maximum air temperature recorded). This represents a substantial increase in leaf temperature compared to OS plants, which experienced considerably lower leaf temperatures during these days. This elevated leaf temperature is likely exacerbated by several factors. Firstly, diminished soil moisture, coupled with the larger size of the OTC plants, likely intensified drought stress. Secondly, reduced transpiration in OTC plants, which typically acts as a cooling mechanism, further contributed to higher leaf temperatures. Finally, the increased boundary layer by larger OTC plants may have also increased the risk of extreme heat events (Figures 3 and 6; Table 1).

This study investigated how the typically benign warming conditions driven by OTCs, when combined with prolonged periods of reduced water availability and heatwave events during this growing season, exacerbated physiological drought and intensified stress in Antarctic vascular plants. Previous research has shown that in situ warming by OTCs can influence photosynthesis in tundra environments (e.g., Oberbauer et al. 2007; Hernández‐Fuentes et al. 2015; Henry et al. 2022), as observed at our experimental site in King George Island after 4 years of warming (Sáez, Cavieres, et al. 2018; Sáez, Galmés, et al. 2018). There, CQ showed higher *A*_N_ driven by increased *g*_m_, which was linked to anatomical changes such as higher mesophyll surface area exposed to mesophyll airspaces per unit leaf area (*S*_m_/*S*) in OTC plants compared to those in OS. This ultimately resulted in higher growth for CQ. In contrast, no differences were observed in DA (Sáez, Galmés, et al. 2018). It has been shown that, in tundra plant species, long‐term responses to warming (with OTC) differ from short‐term responses, whereas for some species, warming‐stimulated growth is evident only after long‐term exposure (Hollister et al. 2005; Elmendorf et al. 2012), which may be the case for DA several years later than the results shown by Sáez, Galmés, et al. (2018). In our field campaign, OTC DA and CQ plants were much bigger than OS plants, with DA showing larger differences between both sites than CQ (Figure 1). The increased size of DA plants within the OTCs would lead to increased transpiration and exacerbate physiological drought compared to OS conditions; moreover, considering that for the central period of the growing season, soil water availability was considerably reduced in the OTCs (Figure S1). Additionally, the exuberant growth of DA plants in OTCs could partially cover CQ, thus restricting incoming radiation, transpiration and drought stress (Bravo, pers. comm.; Figure 1). However, *A*_N_ was much lower than those observed in previous years at the same site. Both OTC and OS plants exhibited a substantial reduction in photosynthesis, with an 85% reduction in *A*_N_ within the OTCs and around a 50% reduction in OS measured at the same temperature (Sáez, Galmés, et al. 2018; Gago et al. 2023). This decrease in *A*_N_ was largely driven by strong diffusive limitations, with DA experiencing the most pronounced limitations. In CQ, however, *g*_s_ and *g*_m_ were within their usual range when compared to previous studies (Sáez, Galmés, et al. 2018; Clemente‐Moreno et al. 2020a, 2020b; Gago et al. 2023). In fact, the diffusive constraints were so severe in DA that, when we measured *A*_N_ at low temperatures by reducing block temperature up to 5°C in the gas‐exchange measuring chamber, it remained unaffected (Table 1). This contrasts with previous studies, which reported significant declines in photosynthesis at suboptimal temperatures, but measuring plants under much better physiological conditions (Xiong et al. 1999; Sáez, Cavieres, et al. 2018; Sáez, Galmés, et al. 2018; Sanhueza et al. 2019, 2022; Clemente‐Moreno et al. 2020a, 2020b).

Significant reductions in *g*_s_ were observed in DA and CQ plants grown inside the OTC. Additionally, across all conditions, both species exhibited extremely low *g*_m_ values (Table 1). Beyond the well‐established role of stomatal closure under drought to prevent dehydration (which imposes CO_2_ limitations on photosynthesis), it is now recognised that *g*_m_ plays a similarly important role, if not greater, than *g*_s_ (Gago et al. 2014; Flexas et al. 2016; Nadal and Flexas 2018). Data on similar behaviour in tundra species remain scarce, though recent studies on DA and CQ grown under various conditions over the past decade consistently suggest that *g*_m_ is the primary limitation for both species (Sáez, Cavieres, et al. 2018; Sáez, Galmés, et al. 2018; Clemente‐Moreno et al. 2020a, 2020b; Gago et al. 2023). It has been proposed that the strong mesophyll limitation is, at least, partially offset by efficient photobiochemistry, such as the high Rubisco specificity factor observed in both species at low temperatures (Sáez, Galmés, et al. 2018). The mechanisms behind the restricted *g*_m_ dynamics appear to be linked to xerophytic leaf anatomy traits, including increased LMA due to dense mesophyll cell packing, which reduces intercellular air spaces, and thicker cell walls (Sáez, Cavieres, et al. 2018). Critically, a significant knowledge gap persists regarding the biochemical mechanisms governing mesophyll conductance (*g*_m_) in these Antarctic species. Specifically, the roles of key drivers such as aquaporins, carbonic anhydrases, and cell wall and membrane composition remain largely unexplored, which could provide insights into the regulation of *g*_m_ and adaptation to extreme environments (Gago et al. 2020; Lundgren and Fleming 2020; Evans 2021; Mizokami et al. 2022).

An often overlooked aspect of growth and photosynthesis changes induced by passive warming methods like OTCs is their potential impact on plant stress tolerance. The more benign conditions driven by OTCs could lead to increased carbon assimilation and water use efficiency by anatomical improvements (Sáez, Cavieres, et al. 2018), but those rearrangements could reduce leaf dehydration tolerance as previously observed (Gago et al. 2014; López‐Pozo et al. 2019; Nardini 2022), (Perera‐Castro and Flexas 2023; Quintanilla et al. 2023; Nadal et al. 2023; Carriquí et al. 2025). In our study, DA plants grown under OTC conditions exhibited a significant reduction in LMA compared to those observed in OS (Table S1), suggesting that leaf development occurred under past benign conditions in the passive warming chamber. However, a lower LMA could also indicate reduced tolerance to dehydration (Nardini 2022), as LMA is a function of leaf thickness and density, with up to 70% of density explained by cell wall thickness and composition (Onoda et al. 2017), which drives the cell wall elastic modulus (*ε*) (Bartlett et al. 2012). The leaf *ε* is essential to define a major physiological leaf trait for drought tolerance: the leaf water potential at the turgor loss point (Ψ_tlp_). This trait basically sets the threshold at which cells lose turgor pressure, increasing the risk of plasmolysis, cell collapse and death (Nardini 2022). The lower the Ψ_tlp_, the greater the capacity to tolerate dehydration without cell collapse. Species from dry environments tend to present lower Ψ_tlp_ values accompanied by higher *ε* (Nadal et al. 2018; Petruzzellis et al. 2021). Our results agree with this, showing reduced leaf dehydration tolerance in DA under OTC conditions, with a significant 17% reduction in LMA. In contrast, CQ exhibited no significant changes in LMA or leaf dehydration tolerance, neither under OTC nor OS (Tables S1 and 2).

In addition to structural leaf traits, dehydration tolerance also depends on osmotic agents, cell wall, membrane, and protein‐specific osmoprotectants and antioxidant capacity to mitigate oxidative stress (Oliver et al. 2010). For this purpose, we analysed the primary metabolic profile of DA and CQ grown in OTCs and OS and observed several differences between the conditions for both species (Figures 4 and 5). In DA, tryptophan levels were increased in individuals grown in OTCs (under severe physiological stress) compared to those from OS (Table 1; Figure 5). The higher levels observed in this metabolite could indicate the activation of secondary metabolism antioxidant routes; particularly, the pathway leading to the production of flavonoids and phenylpropanoids, compounds that are known to enhance antioxidant capacity (Zhao et al. 1998; Hoffmann et al. 2004). We previously observed constitutive, high antioxidant activity in DA grown at 5°C and 15°C, although these plants did not show drastic changes in tryptophan content (Clemente‐Moreno et al. 2020b), which could indicate that this route is not activated by low temperatures. DA plants grown in OTCs also had higher levels of soluble sugars (glucose, fructose, sucrose and trehalose) and sugar alcohols (glycerol, mannitol, galactinol and *myo*‐inositol) than those from OS (Figure 5). These compounds have been associated with protein and membrane stabilisation. We also observed increased levels of xylose, a component of hemicellulose and indicative of cell wall remodelling, which in turn could improve temperature stress tolerance (Tenhaken 2015; Panter et al. 2020; Takahashi et al. 2021). Additionally, the intermediates of the TCA cycle (citrate, isocitrate, fumarate and malate) displayed higher levels in DA from OTCs than those from OS (Figure 5). Overall, these metabolic changes are similar to those observed when comparing DA plants grown under limited nutrient conditions with individuals from nitrogen‐rich environments (Gago et al. 2023).

We speculate that these molecular responses could improve the tolerance of DA to several stress types, such as drought, heat and low/freezing temperatures (Sui et al. 2007; Upchurch 2008; Kwon et al. 2012; Obata and Fernie 2012; Van den Ende 2013; Wang et al. 2022). Indeed, all these stressful conditions were present during the growing season at our experimental site, although they were more severe within the OTCs (Figures 2, 3 and S1). These results align well with the higher physiological stress observed in DA individuals growing under such conditions (Table 1). The accumulation of soluble sugars could also indicate changes in carbon allocation, as growth inhibition redirects carbon flow from structural or storage compounds to soluble sugars (Kaur et al. 2021). Although soluble sugars can play an osmotic role, they were insufficient to significantly reduce the Ψ_tlp_ and prevent cell collapse during the most severe dehydration conditions (silica gel) used in the leaf dehydration test, where leaves from the OTC plants exhibited lower tolerance than OS leaves (Table 2; Figure 6). Thus, it seems that this metabolic profile could alleviate oxidative stress under OTC conditions, but it is not enough to compensate for LMA reductions, thus diminishing dehydration tolerance. Further studies are needed to elucidate whether osmotic potential and/or modulus of elasticity may play an important role in driving dehydration tolerance in these species in Antarctica.

In CQ, proline and fumaric acid displayed the highest VIP scores in the PLS‐DA analysis, although with different trends (Figure 4d), and their levels were significantly different in plants from OTCs and OS (Figure 5). Unlike DA, no physiological stress differences were observed between CQ individuals in OTC compared to OS, as suggested by the physiological indicator ETR/*A*_N_ (Perera‐Castro and Flexas 2023) (Table 1). However, reductions in *g*_s_, ETR and *P*_r_ were in line with the lower soil water content in the OTCs compared to OS (Figure 3; Table 1). The reduced levels of proline in CQ from OTCs compared to OS plants could be attributed to its multifunctional role under varying environmental conditions, either as an osmolyte, radical scavenger or energy source once the stress is relieved (Kavi Kishor and Sreenivasulu 2014; Gago et al. 2017; Fernández‐Marín, Nadal, et al. 2020). As previously mentioned, fumarate exhibited the opposite trend, with significant increases in CQ from OTCs compared to plants from OS (Figure 5). This metabolite, an intermediate of the TCA cycle, serves as an additional source of carbon skeletons for various biosynthetic pathways, besides its role as a precursor of malate (Zell et al. 2010; Arnold and Nikoloski 2014). Both fumarate and malate are strongly linked to the redox shuttling between organelles, balancing the ATP/NADPH ratio and energy supply, key processes during stress (Araújo et al. 2011; Dinakar et al. 2016; Wang et al. 2016). Fumarate has recently been proposed as a metabolic fail‐safe mechanism, ensuring constant malate accumulation under varying environmental conditions and supporting photosynthetic acclimation to both low and high temperatures (Saunders et al. 2022). The TCA cycle also provides oxaloacetate for methionine synthesis in the chloroplast. Notably, methionine was one of the 10 VIP metabolites in the PLS‐DA analysis, with the same trend as fumarate (increased). This phenomenon has also been observed in CQ at low temperatures in previous studies (Clemente‐Moreno et al. 2020a).

## 5 Conclusions

OTCs have a significant impact on the micro‐environment and physiological responses of Antarctic vascular plants. Previous findings indicate that while OTCs provide a beneficial warming effect, they could also –depending on the growing season weather conditions– exacerbate drought and heat stress by increasing VPD and lowering soil moisture. This would lead to specific events of high leaf temperature that could not be cooled by transpiration. In this sense, we observed that, from the two Antarctic vascular species, DA showed the most significant alterations through reductions in *g*_s_ and *g*_m_ that extremely constrained photosynthesis, accompanied by a severe physiological stress response. Additionally, DA plants grown in OTCs were the most vigorous individuals, although with a marked reduction in LMA, probably driven by warmer conditions at the beginning of the growing season. This structural acclimation to a benign environment may explain its reduced stress tolerance compared to plants grown at OS. The primary metabolic profile of DA from OTCs showed known drought and heat stress responses, mainly driven by the accumulation of secondary metabolite precursors (tryptophan and nicotinate), osmolytes and stabilisers of macromolecules (such as glucose, fructose and trehalose, as well as glycerol and myo‐inositol) and cell wall‐related metabolites (xylose). These changes could enhance antioxidant capacity, structure stabilisation and osmoprotection under drought and heat stress to ultimately alleviate oxidative stress; nevertheless, they may not compensate for the reduced stress tolerance associated with the lower LMA of OTC plants compared to those grown at OS. CQ, on the other hand, exhibited fewer physiological, structural and metabolic differences between OTC and OS, likely due to its smaller size compared to DA, which in turn reduced its water requirements. Overall, our results indicate that further studies are needed to better understand the physiological and metabolic responses of Antarctic vascular plants to the predicted future warming and drying conditions.

## Author Contributions

G.J. and B.L.A. planned and designed the research. G.J., B.L.A., and C.M. conducted fieldwork and performed the experiments. G.J., A.M., and F.C.M. analysed data. G.J., C.M., A.M., N.‐N.A., F.C.M., F.A.R., C.‐M.M.J., G.J., F.J., C.L.A., and B.L.A. wrote the manuscript.

## Supporting information


Data S1.

## Data Availability

Data available on request from the authors.
