# Supplementary material for: A Double Edge‐Sword in Antarctica: In Situ Passive Warming Exacerbates Drought and Heat Stress Differentially in the Native Vascular Species
Source: Physiol Plant. 2025 Jul 14;177(4):e70399. doi: 10.1111/ppl.70399 (PMC12257275; doi:10.1111/ppl.70399)
Supplement: Supplementary file 1 — Data S1. [file PPL-177-e70399-s001.pdf]

SUPPLEMENTALS

A double edge-sword in Antarctica: *in situ* passive warming exacerbates drought and heat stress differentially in the native vascular species

Gago Jorge[1,*], Carriquí Marc[1], Ayuso Manuel[1], Nunes-Nesi Adriano[2], Figueroa Carlos María[3], Fernie Alisdair Robert[4], Clemente-Moreno María José[1], Gulías Javier[1], Flexas Jaume[1], Cavieres Lohegrin Alexis[5], Bravo León Aloys[6]

[1]Agro-Environmental and Water Economics Institute (INAGEA), Research Group of Plant Biology under Mediterranean Conditions, Department of Biology, Universitat de les Illes Balears, Palma, Spain

[2]National Institute of Science and Technology on Plant Physiology under Stress Conditions, Departamento de Biologia Vegetal, Universidade Federal de Viçosa, 36570-900 Viçosa, MG, Brazil

[3]Instituto de Agrobiotecnología del Litoral, UNL, CONICET, FBCB, Santa Fe, Argentina

[4]Central Metabolism Group, Molecular Physiology Department, Max-Planck-Institut für Molekulare Pflanzenphysiologie, Golm, Germany

[5]Departamento de Botánica, Facultad de Ciencias Naturales y Oceanográficas, Universidad de Concepción and Instituto de Ecología y Biodiversidad (IEB), Concepción, Chile

[6]Laboratorio de Fisiología y Biología Molecular Vegetal, Dpt. de Cs. Agronómicas y Recursos Naturales, Facultad de Cs. Agropecuarias y Medioambiente, Instituto de Agroindustria, Universidad de La Frontera, Temuco, Chile

***Correspondence**
Jorge Gago
Email: jorge.gago@uib.cat

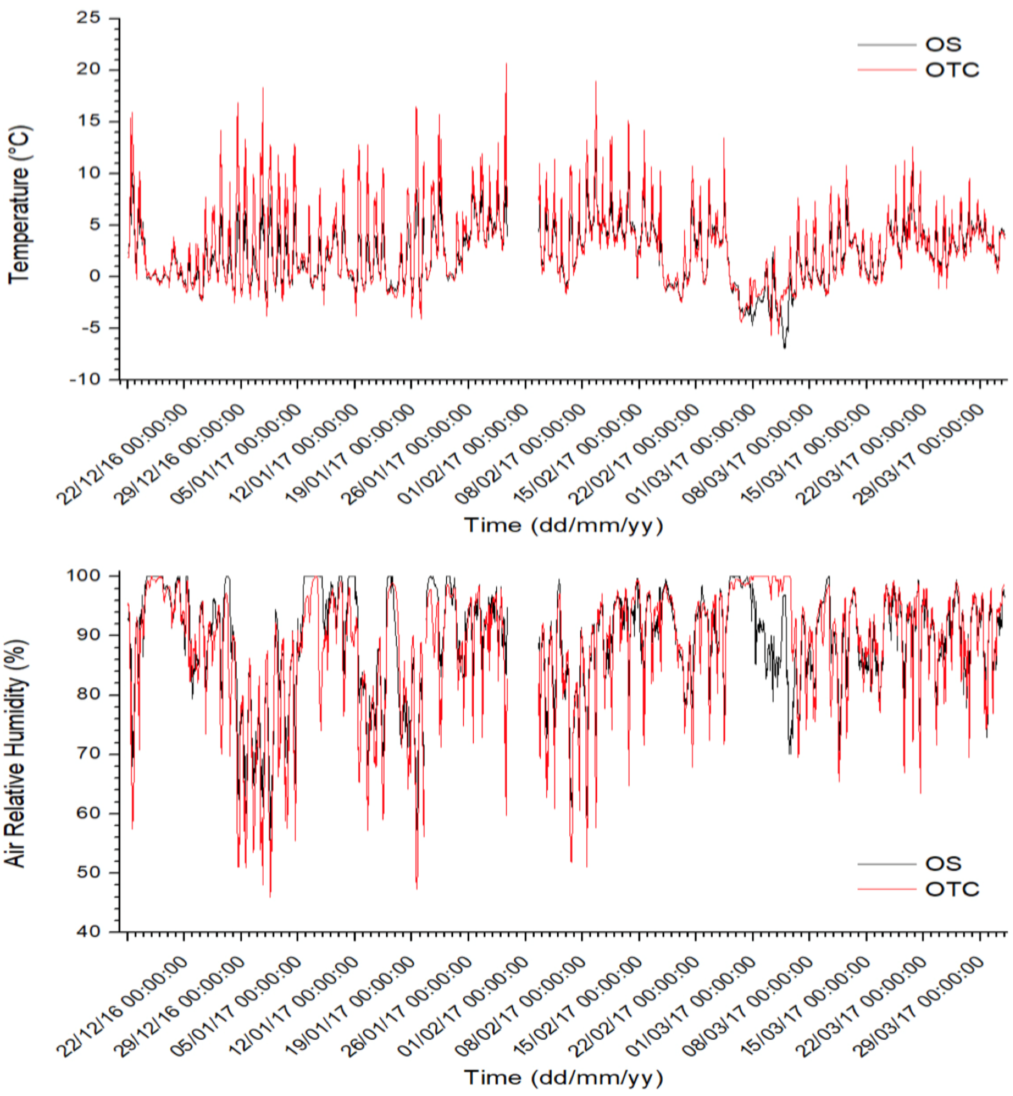

Fig. S1. Air temperature and relative humidity (RH) recorded throughout the 2016-2017 growing season, comparing conditions inside the open-top chamber (OTC) and outside (OS).

Table S1. Leaf absorbance, leaf dry mass per area (LMA) and relative water content (RWC) for *Deschampsia antarctica* (DA) and *Colobanthus quitensis* (CQ) individuals growing in open space (OS) and inside open-top chambers (OTC). Values are presented as means ± SE (n = 10-15 for LMA, n = 8 for RWC and absorbance n =3-5). Significant differences between treatments ($p < 0.05$) are indicated with an asterisk. F and p-values are provided for each comparison between treatments.

Species	Treatment	Leaf absorbance (unitless)	LMA (g m^{-2})	RWC (%)
**DA**	**OS**	94.0 ± 4.3	91.9 ± 4.5	96.8 ± 1.2
	**OTC**	98.1 ± 0.1	76.9 ± 2.8*	93.9 ± 2.1
	F	1.71	7.35	1.39
	p-value	0.238	0.011	0.26
**CQ**	**OS**	98.5 ± 0.4	86.5 ± 6.0	85.5 ± 2.6
	**OTC**	98.6 ± 0.1	86.7 ± 3.4	93.6 ± 1.3*
	F	0.08	0	7.74
	p-value	0.932	0.97	0.01

Table S2. Average metabolite peak areas normalized by ribitol and dry weight (± SE) for each species (*Deschampsia antarctica* and *Colobanthus quitensis*; DA and CQ, respectively) and growth conditions (open-top chamber and open space; OTC and OS, respectively). Significant differences between growth conditions within the same species are indicated by asterisks (t-test, p < 0.05).

	OSCQ	OTCCQ	OSDA	OTCDA
Alanine	0.0202 ± 0.0011	0.0208 ± 0.0009	0.0184 ± 0.0004	0.0197 ± 0.0028
Pyruvic acid	0.0002 ± 0.0000	0.0002 ± 0.0000	0.0001 ± 0.0000	0.0001 ± 0.0000
Valine	0.0202 ± 0.0013	0.0197 ± 0.0019	0.0102 ± 0.0011	0.0092 ± 0.0011
Glycerol	0.0180 ± 0.0017	0.0286 ± 0.0040*	0.0167 ± 0.0022	0.0241 ± 0.0017*
Isoleucine	0.0073 ± 0.0006	0.0067 ± 0.0006	0.0039 ± 0.0004	0.0034 ± 0.0004
Glycine	0.0174 ± 0.0020	0.0178 ± 0.0019	0.0039 ± 0.0005	0.0057 ± 0.0010
Phosphoric acid	0.0810 ± 0.0177	0.0513 ± 0.0044	0.0042 ± 0.0006	0.0048 ± 0.0014
Proline	0.3651 ± 0.0171	0.1099 ± 0.0079*	0.0311 ± 0.0034	0.0222 ± 0.0048
Urea	0.0006 ± 0.0001	0.0008 ± 0.0001	0.0002 ± 0.0000	0.0005 ± 0.0001*
Glyceric acid	0.0118 ± 0.0007	0.0138 ± 0.0015	0.0047 ± 0.0007	0.0068 ± 0.0009
Benzoic acid	0.0002 ± 0.0000	0.0002 ± 0.0000	0.0001 ± 0.0000	0.0001 ± 0.0000
Serine	0.0234 ± 0.0015	0.0196 ± 0.0009*	0.0091 ± 0.0011	0.0078 ± 0.0011
Succinic acid	0.0187 ± 0.0008	0.0187 ± 0.0010	0.0026 ± 0.0001	0.0029 ± 0.0004
Threonine	0.0074 ± 0.0005	0.0076 ± 0.0006	0.0040 ± 0.0004	0.0036 ± 0.0005

Fumaric acid	0.0046 ± 0.0006	0.0075 ± 0.0006*	0.0009 ± 0.0001	0.0017 ± 0.0002*
Nicotinic acid	0.0007 ± 0.0000	0.0007 ± 0.0001	0.0004 ± 0.0000	0.0005 ± 0.0000*
Alaninebeta	0.0006 ± 0.0001	0.0004 ± 0.0000*	0.0001 ± 0.0000	0.0002 ± 0.0000
Homoserine	0.0007 ± 0.0000	0.0007 ± 0.0000	0.0003 ± 0.0000	0.0005 ± 0.0001
Erythritol	0.0003 ± 0.0000	0.0003 ± 0.0000	0.0001 ± 0.0000	0.0002 ± 0.0000
Malic_acid	0.0654 ± 0.0040	0.0645 ± 0.0020	0.0224 ± 0.0022	0.0409 ± 0.0037*
Butyric_acid4-amino	0.0194 ± 0.0041	0.0320 ± 0.0052	0.0140 ± 0.0017	0.0173 ± 0.0022
Aspartic acid	0.0163 ± 0.0016	0.0141 ± 0.0020	0.0026 ± 0.0002	0.0024 ± 0.0003
Threonic acid	0.0221 ± 0.0009	0.0179 ± 0.0018	0.0067 ± 0.0009	0.0100 ± 0.0009*
Methionine	0.0007 ± 0.0000	0.0011 ± 0.0001*	0.0001 ± 0.0000	0.0001 ± 0.0000
Asparagine	0.0002 ± 0.0000	0.0003 ± 0.0000	0.0001 ± 0.0000	0.0001 ± 0.0000
Glutamine	0.0020 ± 0.0002	0.0022 ± 0.0004	0.0002 ± 0.0000	0.0001 ± 0.0000
Xylose	0.0022 ± 0.0002	0.0034 ± 0.0004*	0.0017 ± 0.0001	0.0027 ± 0.0004*
Glutamic acid	0.0394 ± 0.0033	0.0379 ± 0.0013	0.0066 ± 0.0006	0.0057 ± 0.0010
Malonic acid	0.0001 ± 0.0000	0.0001 ± 0.0000	0.00005 ± 0.00000	0.0001 ± 0.0000*
Putrescine	0.0003 ± 0.0000	0.0003 ± 0.0000	0.0008 ± 0.0001	0.0007 ± 0.0001
Phenylananine	0.0036 ± 0.0005	0.0049 ± 0.0004	0.0032 ± 0.0003	0.0032 ± 0.0004
Asparagine	nd	nd	0.0005 ± 0.0001	0.0007 ± 0.0001
Ornithine	0.0004 ± 0.0001	0.0004 ± 0.0001	0.0001 ± 0.00001	0.0001 ± 0.00002
Glyceraldehide_3_phosphate	0.0012 ± 0.0002	0.0012 ± 0.0001	nd	nd
Dehydroascorbic_dime	0.0042 ± 0.0010	0.0058 ± 0.0016	0.0032 ± 0.0003	0.0048 ± 0.0004*
Tyramine	0.0002 ± 0.0000	0.0002 ± 0.0000	0.0005 ± 0.0001	0.0006 ± 0.0000
Inositol_myo	0.0543 ± 0.0037	0.0565 ± 0.0029	0.0048 ± 0.0007	0.0084 ± 0.0012*
Tyrosine	0.0010 ± 0.0003	0.0023 ± 0.0005	0.0008 ± 0.0001	0.0013 ± 0.0002
Cinnamic_acid_4_hydroxy	0.0003 ± 0.0000	0.0003 ± 0.0000	0.00013 ± 0.00002	0.00020 ± 0.00002*
Inositol_1_phospate	0.0018 ± 0.0002	0.0023 ± 0.0002*	0.00054 ± 0.00012	0.00096 ± 0.00016
Tryptophan	0.0002 ± 0.0000	0.0002 ± 0.0000	0.00004 ± 0.00000	0.00021 ± 0.00001*
Galactinol	0.1961 ± 0.0210	0.2515 ± 0.0251	0.0042 ± 0.0006	0.0108 ± 0.0023*
Ribose	0.1380 ± 0.0045	0.1263 ± 0.0052	0.0997 ± 0.0007	0.1081 ± 0.0085
Fructose	1.0837 ± 0.2906	1.2480 ± 0.3331	1.5172 ± 0.0173	2.5339 ± 0.2544*
Mannitol	0.8113 ± 0.2187	1.0364 ± 0.3070	1.3728 ± 0.0255	2.2507 ± 0.2224*
Galactose	0.2122 ± 0.0173	0.1846 ± 0.0138	nd	nd

Citric acid	0.2384 ± 0.0116	0.2602 ± 0.0327	0.0327 ± 0.0032	0.0650 ± 0.0055*
Isocitric acid	0.0130 ± 0.0019	0.0136 ± 0.0022	0.0068 ± 0.0004	0.0112 ± 0.0006*
Glucose_D	0.0804 ± 0.0103	0.1226 ± 0.0161	0.3672 ± 0.0179	0.6558 ± 0.0325
Sucrose	2.2230 ± 0.1141	1.9160 ± 0.1154	1.0550 ± 0.0250	1.5833 ± 0.1731*
Trehalose	0.0014 ± 0.0001	0.0016 ± 0.0001	0.0013 ± 0.0001	0.0035 ± 0.0003*
Raffinose	1.8044 ± 0.1204	1.7562 ± 0.1070	0.1475 ± 0.0012	0.1512 ± 0.0175
